# The effects of insects, nutrients, and plant invasion on community structure and function above-and belowground

**DOI:** 10.1002/ece3.961

**Published:** 2014-02-17

**Authors:** Phoebe Wright, Melissa A Cregger, Lara Souza, Nathan J Sanders, Aimée T Classen

**Affiliations:** 1 Department of Ecology and Evolutionary Biology, University of Tennessee569 Dabney Hall, Knoxville, Tennessee, 37996; 2 Institute for Genomic Biology, University of Illinois1206 W Gregory Rm 3405, Urbana, Illinois, 61801; 3 Oklahoma Biological Survey and Department Microbiology and Plant Biology, University of Oklahoma111 E Chesapeake St., Norman, Oklahoma, 73019; 4 Center for Macroecology Evolution and Climate, Natural History Museum of Denmark, University of CopenhagenCopenhagen, DK-2100, Denmark

**Keywords:** aboveground biomass, bacteria, fungi, insect, invasive plant, nitrogen-fixer, nutrient amendment, old-field ecosystem, soil enzyme activity

## Abstract

Soil nutrient availability, invasive plants, and insect presence can directly alter ecosystem structure and function, but less is known about how these factors may interact. In this 6-year study in an old-field ecosystem, we manipulated insect abundance (reduced and control), the propagule pressure of an invasive nitrogen-fixing plant (propagules added and control), and soil nutrient availability (nitrogen added, nitrogen reduced and control) in a fully crossed, completely randomized plot design. We found that nutrient amendment and, occasionally, insect abundance interacted with the propagule pressure of an invasive plant to alter above-and belowground structure and function at our site. Not surprisingly, nutrient amendment had a direct effect on aboveground biomass and soil nutrient mineralization. The introduction of invasive nitrogen-fixing plant propagules interacted with nutrient amendment and insect presence to alter soil bacterial abundance and the activity of the microbial community. While the larger-scale, longer-term bulk measurements such as biomass production and nutrient mineralization responded to the direct effects of our treatments, the shorter-term and dynamic microbial communities tended to respond to interactions among our treatments. Our results indicate that soil nutrients, invasive plants, and insect herbivores determine both above-and belowground responses, but whether such effects are independent versus interdependent varies with scale.

## Introduction

Decades of research demonstrate that soil nutrient availability, invasive plants, and insect abundance can alter the structure of aboveground communities (Mack et al. 2000; Suding et al. 2005; Ehrenfeld 2010; Fisher et al. 2013). In general, nitrogen (N) limitation increases net primary productivity, decreases plant diversity, and thus alters community and ecosystem structure (Cleland and Harpole 2010). Similarly, the invasion of non-native plants in a community can reduce plant diversity and increase plant biomass by more than 50% relative to uninvaded communities (Vila et al. 2011). Insect presence, on average, leads to around a 13% reduction in NPP and causes changes in plant community structure sometimes promoting while other times hindering plant diversity (Hunter 2001; Coupe and Cahill 2003). While it is clear that main and interactive effects of invasive plant species, insect abundance, and soil nutrient availability can shape aboveground plant community structure and function, fewer studies have examined how these factors, solely or in concert, affect the belowground components of ecosystems (Bardgett and Wardle 2010).

Although the above-and belowground compartments of ecosystems are often linked (Wardle et al. 2004), belowground responses to these key factors may not simply mirror aboveground responses (Van Der Putten et al. 2001; Wardle et al. 2004). Independently, invasive plants can change the abundance and type of resources made available to the soil microbial pool and thus alter both the composition of microbial communities and associated functions such as microbial enzymatic activity and respiration (Hollinger 1986; Kourtev et al. 2002). Additionally, via their effects on plant quality and production, insects can alter soil communities and their function by selectively consuming plant material, altering plant chemistry, or by altering frass and waste inputs (Hunter 2001; Frost and Hunter 2004; Classen et al. 2006). Finally, changes in soil nutrient availability can alter soil microbial community composition and function, either by directly altering resources made available for mineralization by belowground communities, or by indirectly altering plant community composition, which may shift the quality and quantity of plant inputs to the soil (Waldrop et al. 2004). Numerous studies show that the addition of nutrients to the soil can shift the composition of the microbial community (Ramirez et al. 2010; Zechmeister-Boltenstern et al. 2011) potentially altering microbial function as well. Overall, invasive species, nutrients, and insect presence can alter belowground community structure and function affecting associated ecosystem processes.

Few studies, to our knowledge, have examined how these three key drivers of plant community structure and ecosystem function – invasive plant species, aboveground insects, and soil nutrient availability – interact to shape both above-and belowground community structure, function, and associated ecosystem processes. In this study, we examined the main and interactive effects of these drivers on above-and belowground community structure and ecosystem function after 6 years of experimentally manipulating the abundance of invasive plant propagules entering the system (at two levels), insect presence (at two levels), and soil nitrogen availability (at three levels) in an old-field ecosystem in the eastern United States. Previous work at this site found that soil nutrient availability interacted with propagule supply to alter the production of an invasive nitrogen-fixing plant (Sanders et al. 2007). When herbivores and nutrients were reduced, the biomass of a nitrogen-fixing shrub increased (Sanders et al. 2007). Given plant biomass production, which is influenced by propagule addition, insect abundance, and nutrient amendments can directly and interactively alter soil microbial communities, it is difficult to predict how belowground communities will respond to our treatments. Broadly, we predict that nutrient amendment will have a large and direct effect on plant production and nutrient mineralization increasing carbon inputs into the soil system thus influencing belowground community structure and function. Consequently, the interactive effects of invasive propagules and insects will influence fine-scale belowground community structure and function in the context of large carbon inputs.

## Methods

**Figure 1:**
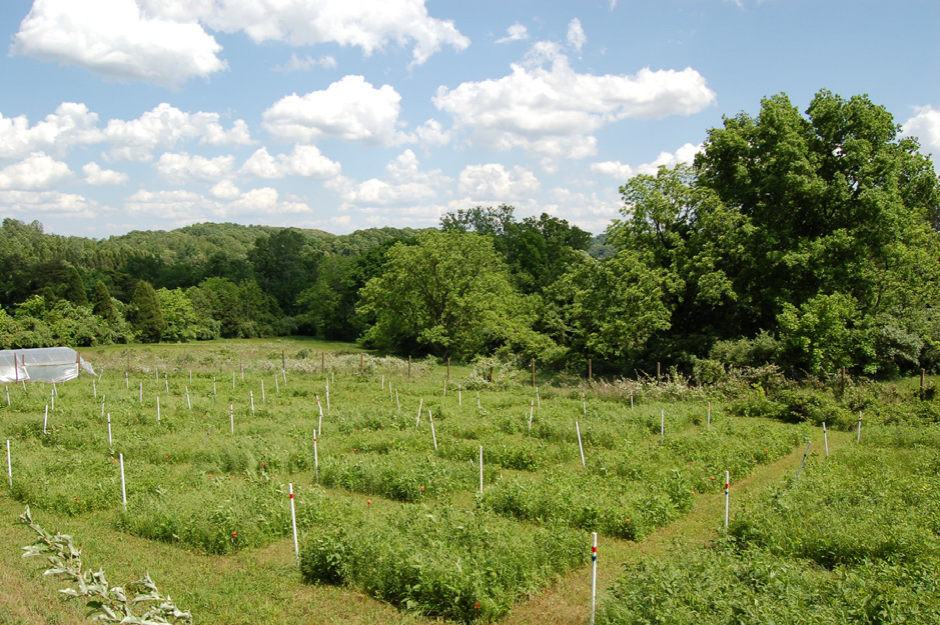
The experimental field site located near Oak Ridge, Tennessee, USA. We manipulated the density of invasive plant propagules, insect abundance, and soil nutrient availability in a fully crossed, completely randomized design.

In 2004, we established a multifactor experiment in an intact old field located on the Oak Ridge National Environmental Research Park, Tennessee (35°58′N 84°17′W). The site was abandoned from agricultural use in 1943 and has been managed since 2003 with an annual mowing regime. Soil at the site is Typic Hapludult, which has a silty clay-loam texture, is slightly acidic, and drains moderately well (Phillips et al. 2001). The three most common plant species, *Verbesina occidentalis*,*Verbesina virginica*, and *Solidago altissima*, make up ∼40% of the plant biomass, and there are approximately 60 other subdominant herbaceous, graminoid, and woody native and exotic species, including *Lespedeza cuneata* (Sanders et al. 2007; Blue et al. 2011; Souza et al. 2011a,b2011b) at the site. In 2004, we erected 3-m-tall deer fence around the site and established 72 3 × 3 m plots, including a 0.5-m buffer around each plot, at a spacing of 2 m among plots, within existing vegetation at the site. Each spring, we manipulated the density of invasive plant propagules, insect abundance, and soil nutrient availability in a fully crossed, completely randomized design (Fig. 1; *n* = 6 for each treatment combination).

From 2004 to 2006, we manipulated the propagules of an invasive nitrogen-fixing plant, *Lespedeza cuneata*, by adding 1700 seeds·m^−2^ with a seed spreader to 36 randomly selected plots (Ernst Conservation Seeds, Meadville, PA). The density of *L. cuneata*, a Rank 1 invasive species, was ∼6 × higher in the seed addition plots than in nonaddition plots (Sanders et al. 2007).

From 2004 to 2009, we manipulated insect presence and absence by applying permethrin insecticide (Hi-Yield Kill-A-Bug, Voluntary Purchasing Group, Bonham, TX) to 36 randomly selected plots. Using a backpack sprayer, 0.23 L·m^−2^ of insecticide was added every 2–3 weeks to insect-removal plots during the growing season. Permethrin, a synthetic pyrethroid-based insecticide, is widely used in ecological studies (e.g., Root 1996; Schmitz et al. 2006). Using a combination of sweep netting, vacuum sampling, and visual scanning, we found that insect abundance was on average 4 × lower in the insect-reduced plots (6.6 individuals m^−2^) relative to the control plots (28.4 individuals m^−2^; Sanders et al. 2007). Based on observations at the field site since 2004 (Lane 2006; Sanders et al. 2007; Crutsinger et al. 2013) and detailed studies on plant–insect interactions conducted at the site (Crawford et al. 2007; Crutsinger et al. 2013), we are confident that herbivores were by far the most abundant trophic group. For example, Lane (2006) surveyed the insect community at the site for 2 years using a series of standard techniques and found that herbivores made up >56% of the total abundance of arthropods at the site. Additionally, of the insect taxa that were most frequently detected in the insect-reduced plots, only one was herbivorous – an aphid that proved difficult for us to remove or reduce using the insecticide treatment and vacuum sampler. Finally, several pilot experiments demonstrated that neither plant growth nor NO_3_-N and NH_4_-N in the soil solution differed between insect-reduced and control plots (Sanders et al. 2007). Thus, we are convinced that our use of permethrin insecticide reduced aboveground herbivore loads in the plots and that it had limited, if any, effects on soil nutrient dynamics in the plots.

From 2004 to 2009, we manipulated soil nutrient availability by doing nothing (control), adding nitrogen to increase nitrogen availability, and adding carbon to reduce nitrogen availability. Nitrogen was added as urea fertilizer (20 g·m^−2^·year^−1^), and carbon was applied as sucrose (167 g·m^−2^·year^−1^) (Sanders et al. 2007; Blue et al. 2011; Souza et al. 2011a,b2011b). Sucrose, which is ∼46% carbon, is quickly mineralized by microbial communities; thus, its addition reduced soil nitrogen availability in our plots (Wang et al. 2004; Craine et al. 2007; Sanders et al. 2007). Relative to control plots, soil nitrogen availability (NO_3_-N + NH_4_-N) was 2 × higher in nitrogen-added plots and 5 × lower in carbon-added plots (*P* < 0.0001; Sanders et al. 2007). Urea fertilization increased NO_3_-N and NH_4_-N availability (*P* < 0.0001), whereas carbon fertilization decreased NO_3_-N availability (*P* < 0.0001) but had no effect on NH_4_-N availability (*P* = 0.50; Blue et al. 2011). Nutrient application rates were similar to fertilization rates used in other experiments (Mclendon and Redente 1992; Siemann 1998).

To assess how the plant community responded to the treatments, we harvested aboveground plant biomass at the end of the growing season in 2009. We randomly placed a 0.5 m × 1 m quadrat near the center of each of the 72 plots and clipped all of the aboveground plant biomass to ground level. We separated Lespedeza from the biomass of other species; biomass was oven-dried for approximately 48 h at 60°C and weighed.

To assess the structure and function of the soil community and the soil properties that might influence it, we collected two sets of soil samples (0–10 cm depth, 5 cm diameter) randomly from each plot in July, 2009. Soils collected for molecular analysis (∼15 g) were immediately frozen with liquid nitrogen and stored at −80°C until analysis. Soil samples for other analyses were returned to the laboratory, homogenized by plot, and sieved to 2 mm. We measured the gravimetric water content (GWC) on each plots sample by oven drying ∼20 g of soil at 105°C for ∼48 h. We measured pH on ∼10 g of dry soil with 0.01 mol L^−1^ CaCl_2_ (Robertson et al. 1999).

Bacterial and fungal relative abundance (gene copy number) were measured using qPCR (see Castro et al. 2010). DNA from each sample was extracted using the UltraClean soil DNA isolation kit (MoBio Laboratories, Carlsbad, CA). DNA concentration and purity were evaluated using a microplate reader (Biotek Instruments, Winooski, VT). PCR for bacteria, 16S rRNA, was performed using bacterial primers Eub 338 (ACT CCT ACG GGA GGC AGC AGZ) (Lane 1991) and Eub 518 (ATT ACC GCG GCT GCT GG) (Muyzer et al. 1993). PCR for fungi, 18S rRNA, was performed using fungal primers nuSSU1196F (GGA AAC TCA CCA GGT CCA GA) and nuSSU1536R (ATT GCA ATG CYC TAT CCC CA) (Borneman and Hartin 2000). In each PCR, abundance for bacteria and fungi was quantified by comparing unknown samples to serial dilutions of 16S and 18S rRNA from Escherichia coli and Saccharomyces cerevisiae, respectively. PCR mixtures for both 16S and 18S rRNA amplifications contained 15 *μ*L of SYBR green master mix (Invitrogen by Life Technologies, Carlsbad, California), 5 *μ*mol of the corresponding primer (Eurofins mwg operon Huntsville, Alabama), and 1 *μ*L of DNA diluted 1:10 with sterile water. All reactions were brought to a final volume of 30 *μ*L with sterile water. Amplification protocol for the 16S rRNA gene consisted of an initial denaturing cycle of 95°C for 3 min, followed by 40 cycles of 95°C for 15 sec, 53°C for 15 sec, and 72°C for 1 min. Amplification for the18S rRNA gene consisted of an initial denaturing cycle of 95°C for 3 min, followed by 40 cycles of 95°C for 15 sec, 53°C for 15 sec, and 70°C for 30 sec. We conducted a melting curve analysis on the products upon completion of the PCR cycle to ensure the purity of the amplification product. We measured fluorescence of the products after each cycle using a 96-well Chromo 4 thermocycler (Bio-Rad Laboratories). We present all data as gene copy number per gram of dry soil (Strickland and Rousk 2010).

We assayed microbial activity and function within 48 h of sample collection using methylumbelliferone (MUB)-linked substrates for three soil enzymes important in carbon, nitrogen, and phosphorus degradation: *β*-glucosidase, N-acetylglucosaminidase (nagase), and phosphatase, respectively. Sample suspensions were prepared by adding ∼1 g of soil to 125 mL of 50 mmol L^−1^, pH 5.0, sodium acetate buffer. Soil suspensions were mixed on a stir plate for 2 min, while 96-well plates were prepared with blank wells, substrate controls, soil negative controls, reference standards, and quench controls. All plates were incubated at room temperature in the dark. Nagase and phosphatase reactions were incubated for 0.5 h, while the ß-glucosidase reaction was incubated for 2 h (Saiya-Cork et al. 2002; Sinsabaugh et al. 2005). Reactions were stopped by adding 25 *μ*L of 0.5 mol L^−1^ sodium hydroxide (NaOH). Fluorescence was measured using a Modulus fluorometer (Turner Biosystems, Sunnyvale, CA) at an excitation of 365 nm and an emission of 450 nm. Potential enzymatic activity was reported as nmol·h^−1^·g^−1^.

Potential soil nitrogen mineralization, nitrification, and ammonification were measured using laboratory incubations (Robertson et al. 1999). Collected soils were brought up to field capacity, and an initial sample was immediately extracted with 2 mol L^−1^ KCL. Another sample was incubated at laboratory temperatures in dark 1-quart Mason jars. Each chamber contained a soil sample and a small vial of water to maintain humidity, and each chamber was flushed with air every 3–7 days. After 28 days, we removed the soils from the incubation and extracted them with 2 mol L^−1^ KCL. Extracts were analyzed for NO_3_-N and NH_4_-N on a Westco Smart Chem Auto Analyzer (Westco Scientific, Brookfield, CT). Initial inorganic nitrogen pools were subtracted from the incubated pools to estimate potential nitrogen mineralization.

For each response variable, we analyzed the main and interactive effects of invasive plants, insect presence, and soil nutrient availability using an analysis of variance (ANOVA). Data were log-transformed when they violated the assumptions of ANOVA, but data shown in the text and figures are untransformed means. We used Tukey's HSD means separation test (*α* = 0.05) to identify whether treatment means differed from one another when necessary.

## Results

**Figure 2:**
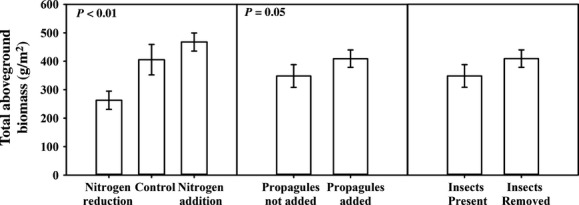
Total aboveground plant biomass (including Lespedeza) was altered by nutrient amendment and the addition of invasive plants. Mean (g m^−2^) aboveground plant biomass (±1 standard error) across all nutrient plots (nitrogen reduction, nitrogen addition, and control), invaded plots (propagules added and propagules not added), and insect abundance plots (insects present and insects reduced).

Total aboveground plant biomass varied threefold among treatments (Fig. 2, Table 1). Total aboveground biomass was 44% higher in nitrogen-added and 35% higher in control plots relative to nitrogen-reduced plots. Aboveground biomass was 15% higher in Lespedeza propagule-added plots than in plots where propagules were not added (Table 1). When we excluded Lespedeza biomass from the total aboveground biomass estimate, the effect of adding invasive propagules on aboveground biomass of the rest of the plant community disappeared (Table 1, *P* = 0.39). Reducing insect abundance did not alter aboveground biomass, and none of the interaction terms were significant.

**Table 1:** Three-way ANOVA testing the main and interactive effects of insect presence, nutrient amendment, and propagule addition on total aboveground biomass including and excluding *Lespedeza cuneata*. Significant *P*-values (<0.05) are in bold, and only significant interaction terms are shown.

	df	SS	*F*-ratio	*P*-value
Total aboveground biomass (with Lespedeza)				
Insect presence	1	0.021	0.437	0.511
Nutrient amendment	2	1.051	10.841	**<0.001**
Propagule addition	1	0.192	3.969	**0.051**
Total aboveground biomass (without Lespedeza)				
Insect presence	1	0.037	0.107	0.7447
Nutrient amendment	2	10.086	14.477	**<0.001**
Propagule addition	1	0.261	0.748	0.3906

**Figure 3:**
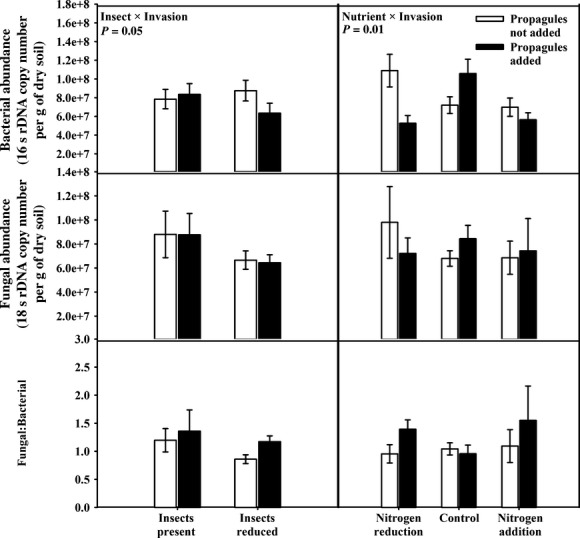
Soil bacterial, but not fungal, abundance (gene copy number) varied threefold among treatments. When insects were present, there was no effect of invasive plant propagules on bacterial abundance, but when insects were reduced, the presence of invasive plant propagules decreased bacterial abundance. Additionally, in nitrogen-reduced plots, the addition of invasive plant propagules reduced bacterial abundance relative to the nitrogen-reduced plots where invasive species were not added. Mean bacterial abundance, fungal abundance, and fungal/bacterial ratio (±1 standard error) across insect abundance plots (insects present and insects reduced) and across nutrient plots (nitrogen reduction, nitrogen addition, and control) and subdivided by plots where propagules were not added (white bars) and where propagules were added (black bars).

Interactions among the treatments altered bacterial abundance, but there were no direct or interactive effects of our treatments on fungal abundance or on the fungal/bacterial ratio (Fig. 3; Table 2). When insect abundances were reduced, the addition of invasive plant propagules lowered bacterial abundance by 28%; however, when insect abundance was not reduced, there was no effect. There was also an interaction between nutrient amendment and invasive plant propagules such that when nitrogen was reduced, the addition of invasive propagules reduced bacterial abundance by 52% relative to the nitrogen-reduced plots where invasive propagules were not added.

**Table 2:** Three-way ANOVA testing the main and interactive effects of insect abundance, nutrient amendment, and propagule addition on bacterial and fungal abundance, the fungal/bacterial ratio, potential soil enzyme activity (*β*-glucosidase, phosphatase, and nagase), and soil potential net nitrogen mineralization, net nitrification, and net ammonification. Significant *P*-values are in bold, and only significant interaction terms are shown.

	df	SS	*F*-ratio	*P*-value
Bacterial abundance (16s rRNA)				
Insect abundance	1	0.041	0.768	0.385
Nutrient amendment	2	0.210	1.987	0.147
Propagule addition	1	0.135	2.554	0.116
Insects × propagules	1	0.209	3.952	**0.052**
Nutrients × propagules	2	0.586	5.550	**0.006**
Fungal abundance (18s rRNA)				
Insect abundance	1	0.044	0.514	0.477
Nutrient amendment	2	0.164	0.970	0.386
Propagule addition	1	0.010	0.117	0.733
Fungal/bacterial				
Insect abundance	1	1.023	1.068	0.306
Nutrient amendment	2	1.202	0.627	0.538
Propagule addition	1	1.287	1.344	0.252
*β*-Glucosidase activity				
Insect abundance	1	2.441	2.491	0.120
Nutrient amendment	2	2.000	1.021	0.366
Propagule addition	1	0.001	0.001	0.974
Nutrients × propagules	2	11.981	6.115	**0.004**
Phosphatase activity				
Insect abundance	1	0.861	0.909	0.344
Nutrient amendment	2	8.887	4.693	**0.013**
Propagule addition	1	4.078	4.306	**0.042**
Nagase activity				
Insect abundance	1	0.107	0.217	0.643
Nutrient amendment	2	2.447	2.479	0.092
Propagule addition	1	0.004	0.008	0.928
Nutrients × propagules	2	2.970	3.010	**0.057**
Potential net ammonification				
Insect abundance	1	0.426	0.250	0.620
Nutrient amendment	2	0.296	0.087	0.917
Propagule addition	1	0.286	0.168	0.685
Potential net nitrification				
Insect abundance	1	0.002	0.019	0.892
Nutrient amendment	2	0.821	4.342	**0.017**
Propagule addition	1	0.115	1.217	0.274
Potential net mineralization				
Insect abundance	1	0.680	1.003	0.321
Nutrient amendment	2	5.652	4.166	**0.021**
Propagule addition	1	0.230	0.339	0.563

**Figure 4:**
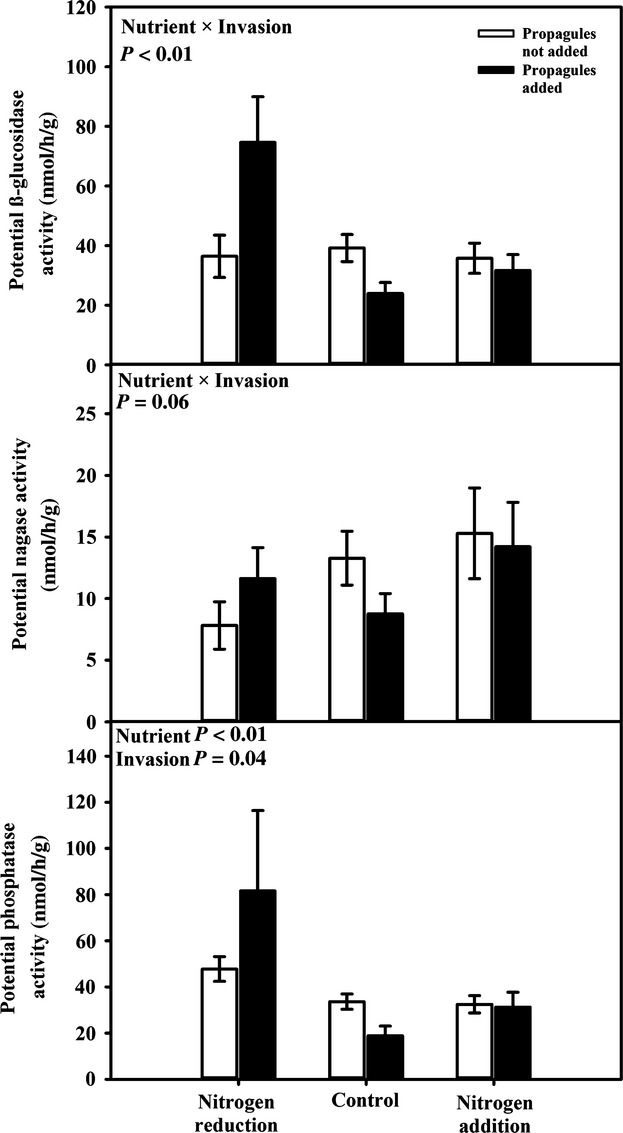
When seeds of Lespedeza were added, *β*-glucosidase activity was 3 × higher in nitrogen-reduced plots than in the control or nitrogen-added plots. Phosphatase activity was 2 × higher in the nitrogen-reduced plots than in the control or nitrogen-added plots, and phosphatase activity was 16% lower in invasive plant propagule-added plots than in the plots where propagules were not added. When nitrogen was reduced, propagule addition increased nagase activity, but propagule addition decreased nagase in control plots. Data shown are mean *β*-glucosidase, N-acetylglucosaminidase (nagase), and phosphatase potential activity (±1 standard error) across nutrient-amended plots (nitrogen reduction, nitrogen addition, and control) and subdivided by plots where propagules were not added (white bars) and where propagules were added (black bars).

Soil microbial *β*-glucosidase activity, which is responsible for releasing glucose molecules for microbial use, varied almost fivefold among treatments (Table 2, Fig. 4). Nutrient amendment and invasive plant propagule addition interacted such that *β*-glucosidase activity was 3 × higher in nitrogen-reduced plots when seeds of Lespedeza were added than in the control or nitrogen-added plots (Table 2, Fig. 4). Phosphatase activity, which removes phosphate groups from a variety of substrates to meet microbial needs for phosphorus, varied fivefold among treatments (Table 2). Phosphatase activity was 2 × higher in the nitrogen-reduced plots than in the control or nitrogen-added plots, and phosphatase activity was 16% lower in invasive plant addition plots than in the plots to which invasive species were not added (Table 2, Fig. 4). Nutrient amendment and the addition of invasive plant propagules interacted to alter soil nagase activity. Propagule addition decreased nagase activity ∼5%, but in nitrogen-reduced plots, propagule addition increased nagase activity.

**Figure 5:**
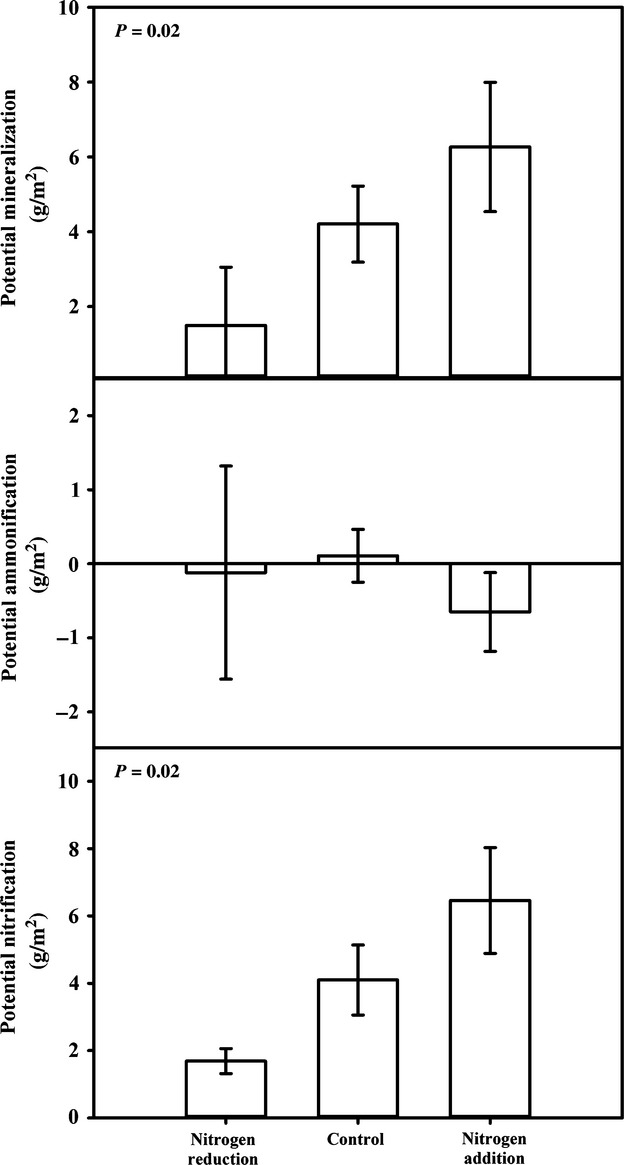
Soil net nitrogen mineralization and nitrification rates were 4 × higher in nitrogen-added plots relative to nitrogen-reduced plots. Data are mean potential mineralization, ammonification, and nitrification (±1 standard error) across nutrient-amended plots (nitrogen reduction, control, and nitrogen addition).

As predicted, the addition of soil nutrients altered potential soil net nitrogen mineralization and nitrification rates. Net mineralization and nitrification rates were 4 × higher in soils from nitrogen-added plots than in soils from nitrogen-reduced plots (Table 2, Fig. 5). There were no effects of either insect reduction or the addition of invasive plant species on net nitrification or net mineralization rates, nor were there any interactive effects. Net ammonification rates did not vary among treatments (Table 2, Fig. 5). Soil gravimetric water content was up to 13% higher in the nitrogen-reduced plots than in the nitrogen-added and control plots (*F*_11,71_ = 1.8, *P* = 0.07). Soil pH did not vary among treatments (*F*_11,71_ = 0.74, *P* = 0.70).

## Discussion

Ecosystem leftacteristics such as nutrient status and insect presence may interact with the presence or abundance of invasive plants to alter ecosystem structure and function. Our study found that nutrient status and, occasionally, insect abundance interacted with the propagule pressure of an invasive plant to alter the above-and belowground structure and function of an old-field ecosystem. Not surprisingly and as predicted, nutrient amendment had a direct effect on aboveground biomass and soil nutrient mineralization; however, the introduction of Lespedeza propagules interacted with nutrient amendment and to a lesser extent insect abundance to alter soil bacterial abundance and the function of the microbial community. Thus, the larger-scale more integrative measures of biomass production and nutrient mineralization appear to be coupled and respond to direct effects of our treatments, while the microbial community, with more rapid turnover times, responds to interactions among our treatments.

As is often the case in other experiments, nutrient availability controlled both aboveground biomass and soil nutrient mineralization in our experiment but in different directions (Vitousek et al. 1997; Pérez et al. 1998; Sirulnik et al. 2007; Cleland and Harpole 2010). Interestingly, adding nitrogen to plots did not control soil nitrogen production or mineralization; both of these processes were different only relative to the nutrient-reduced plots. We suspect that potential nitrification increased as nitrogen was added to plots because the microbial communities in soils exceeded their demand for nitrogen (Zeglin et al. 2007). Eventually, nitrogen saturation (Ågren and Bosatta 1988) was reached resulting in a stagnation of productivity, carbon storage, and increased leaching of nitrogen (Vitousek et al. 1997). Total biomass in our control plots did not differ significantly from that in the nitrogen-added plots indicating that nitrogen saturation may have already occurred in this ecosystem (i.e., these communities were not N-limited). Contrary to other studies that find nitrogen enrichment results in an early increase in ammonification followed by a plateau (Aber et al. 1995) as soil microbial communities become carbon limited (Micks et al. 2004), we found no effects of nitrogen amendments on potential ammonification (Lovett and Rueth 1999; Baron et al. 2000).

Nitrogen reduction decreased aboveground biomass (e.g., carbon input) relative to unamended and nitrogen-added plots, which did not differ from each other. This pattern, where nitrogen addition did not alter aboveground biomass production, was consistent even when we removed Lespedeza biomass from our analysis, indicating that nitrogen reduction lowers production relative to nitrogen addition in control plots irrespective of invasion by Lespedeza. Nutrient reduction at our site also reduced belowground root biomass, indicating that when nutrients were low, plant carbon allocation to the soil and the microbial community which lived there was also low (Blue et al. 2011).

Bacterial abundance was higher when Lespedeza propagules were added to control plots, but lower when propagules were added to plots where nitrogen was reduced. When nitrogen was added, there was no effect of propagule addition on bacterial abundance. These results suggest that in control plots, which were more productive with greater plant inputs than nitrogen-reduced plots, the introduction of nitrogen-fixing Lespedeza propagules reduced nutrient constraints on the bacterial community, thereby increasing bacterial abundance. Since an increase in nitrogen-fixer abundance can alleviate nitrogen constraints on ecosystems, bacterial abundance might also increase (Selmants et al. 2005). However, the stimulating effect of Lespedeza goes away in the less productive nitrogen-reduced plots where plant inputs were lower but carbon addition as sucrose was higher. While we were unable to tease apart exactly how nutrient amendment and the presence of Lespedeza in plots interact to shape soil communities, we have a working hypothesis: changes in plant composition lead to shifts in the structure of soil communities.

Shifts in plant species composition can change carbon allocation to the rhizosphere community leading to shifts in microbial community abundance and composition (Wardle et al. 2003; Innes et al. 2004; Hossain et al. 2010; Ladygina and Hedlund 2010), especially when an ecosystem is invaded by a nitrogen-fixing species (Vitousek and Walker 1989). Because Lespedeza is a nitrogen-fixer, it has a unique community of organisms that occur in its rhizosphere (Kalburtji et al. 2001; Min et al. 2008), thus, as belowground root and exudate abundance from Lespedeza increases relative to other non-nitrogen-fixing species in the community, bacterial abundance might shift (Batten et al. 2008; Yannarell et al. 2011). Previous work in a similar ecosystem found that the presence of Lespedeza altered soil nematode composition relative to other plant species (Kardol et al. 2010), suggesting that the presence of Lespedeza not only changed the bacterial community, but can change the entire soil food web mediating not only bottom-up (e.g., litter phenolics and root exudates) but also top-down (e.g., bacterivorous nematodes) factors influencing soil community structure.

Insects, specifically herbivores, can alter plant inputs into soil communities via their direct impact on plant function (e.g., secondary chemistry and reduced biomass) or by causing a shift in plant community composition (Hillebrand et al. 2007; Gruner et al. 2008). We found that when insects were reduced, bacterial abundance declined 28%, but only when Lespedeza propagules were added. We predicted that decreasing insect abundance would increase carbon allocation belowground, stimulating the microbial community. However, we did not find this pattern. In 2004, Lespedeza biomass was sevenfold higher in plots where insects were removed, propagules were added, and nutrients were reduced (Sanders et al. 2007). Lespedeza litter has high concentrations of phenolic compounds that can retard the growth of microbial communities, and thus, as Lespedeza increases in abundance in insect-reduced plots relative to other plant species bacterial abundance may decrease (Kalburtji et al. 1999, 2001; Min et al. 2008; Yannarell et al. 2011). While we cannot tease apart the presence of Lespedeza and the removal of insects on soil bacterial abundance, they interact in counter-intuitive ways to influence belowground bacterial communities.

While bacterial responses differed among treatments in our experiment, fungal responses did not. In spite of a number of studies showing strong responses of fungi to plant invasion (Van Der Putten et al. 2007; Yannarell et al. 2011), previous work on Lespedeza and invasion by other nitrogen-fixers showed that, relative to bacterial communities, fungal communities were less responsive to Lespedeza presence (Yannarell et al. 2011; St John et al. 2012). In addition, fungal-dominated food webs can take many years to fully develop and are more common in later-successional ecosystems (Wardle 2002). Given the old field we worked in was managed by mowing and was not limited by soil moisture, we suspect that a bacterial-driven energy channel dominated over a fungal energy channel (Witt and Setälä 2010) – a pattern found in other studies examining the presence of nitrogen-fixers in early successional ecosystems (St John et al. 2012).

Changes in bacterial and fungal abundance can indicate that biomass of the community was changing in response to experimental manipulations, but activity and function of that community might not change in tandem (Sinsabaugh et al. 2008). We examined three extracellular enzyme activities that were responsible for the breakdown of organic matter in soils (*β*-glucosidase) or play a key role in soil nitrogen and phosphorus cycling (nagase and phosphatase) (Sinsabaugh et al. 2005). *β*-Glucosidase and nagase activity were higher when propagules of Lespedeza were added to plots where nutrients were reduced, but activities of both enzymes were lower in plots where propagules of Lespedeza were added to plots where nutrients were not manipulated. While there was no statistical interaction between propagule pressure and nutrient amendment on phosphatase activity, the patterns were similar to the other enzymes. The addition of an invasive nitrogen-fixing plant appears to ameliorate the impact of nutrient reduction on soil microbial function. Nitrogen fixation by an invasive plant may have altered the way microbial communities access nutrients in low-nutrient environments, even though this effect did not scale up to alter potential nitrogen mineralization. Similar to our results, Kardol et al. (2010) found that phosphatase activity was lower in soils where Lespedeza was present. We predicted that as nitrogen became less limiting, phosphorus would become more limiting to microbial growth (Demoling et al. 2007). However, when nitrogen was limiting, the addition of Lespedeza increased enzymatic activity across all three enzymes tested. This leads us to infer that nitrogen limitation in the nitrogen-reduced plots was alleviated by the addition of Lespedeza resulting in increased microbial enzyme activity. Similar to our plant and bacterial abundance data, nitrogen addition had no significant effect on any of the enzymes we measured.

Our results indicate that propagule pressure from an invasive nitrogen-fixing plant can interact with soil nutrient status and insect abundance to shape plant and soil communities and potentially the feedbacks between the two. After 6 years of experimental treatments, our larger-scale measurements – plant biomass and soil nitrogen cycling – responded directly to the main effects of our manipulations, while the more fine-scale community measurements tended to be shaped by interactive effects of our treatments, in particular invasive species and nutrient manipulations. Plant–microbe interactions run the gamut from positive interactions that enhance plant growth to negative interactions that slow plant growth (Jackson and Taylor 1996; Oldroyd and Robatzek 2011). Factors such as changes in soil nutrient availability, invasion by non-native plants, and insect abundance can mediate the interactions between plant and soil communities – thus shifting the response of plants and their associated soil communities through time (Ladygina and Hedlund 2010; Fisher et al. 2013). Understanding how above-and belowground components of ecosystems are linked and how those linkages are mediated by invasive plant species, insect abundance, and soil nutrient availability will be increasingly important in a changing world.
